# T2 high-signal-intensity zone of the spinal cord dorsal horn in patients treated with spinal cord stimulation for herpes zoster-associated pain: a retrospective case–control study

**DOI:** 10.1007/s00540-025-03458-1

**Published:** 2025-02-20

**Authors:** Kyosuke Arakawa, Masayuki Nakagawa, Yoichiro Abe, Hiroshi Morimatsu

**Affiliations:** 1 Department of Anesthesiology and Resuscitology, Okayama University Graduate School of Medicine, Dentistry and Pharmaceutical Sciences, 2-5-1 Shikata-Cho, Kita-Ku, Okayama, 700-8558 Japan; 2 Department of Pain Management Clinic, NTT Medical Center Tokyo, Tokyo, Japan

**Keywords:** Herpes zoster, Magnetic resonance imaging, Postherpetic neuralgia, Refractory zoster-associated pain, Temporary spinal cord stimulation

## Abstract

**Purpose:**

In patients with herpes zoster-associated pain (ZAP), magnetic resonance imaging (MRI) has revealed T2 high-signal intensity zones (MRI T2 HIZ) in the dorsal horn of the spinal cord, associated with postherpetic neuralgia (PHN). We retrospectively analyzed the relationship between PHN and MRI T2 HIZ in patients with refractory ZAP in the subacute phase who underwent temporary spinal cord stimulation therapy (tSCS).

**Methods:**

This single-center, case–control study included patients who underwent tSCS for refractory ZAP between 2010 and 2018. MRIs were re-assessed for the presence of T2 HIZ in the dorsal horn of the spinal cord. Patients were divided into T2 HIZ( +) and T2 HIZ(−) groups. Patients with a numerical rating score (NRS) ≥ 3 at the last visit were defined as PHN. The NRS values and the incidence rate of PHN were compared between the two groups.

**Results:**

Of the 67 cases extracted, 38 were included in the analysis: 22 in T2 HIZ( +) group and 16 in T2 HIZ(−) group. No significant differences were observed in background factors between the two groups. However, the T2 HIZ( +) group had a significantly higher NRS at the final visit (T2 HIZ( +):3.8 ± 2.1, T2 HIZ(−):1.4 ± 1.5; P < 0.05) and had significantly more patients with PHN than the T2 HIZ(−) group (T2 HIZ( +) vs. T2 HIZ(−), 15/22 (68%) vs. 3/16 (19%); odds ratio = 8.67; 95% confidence interval, 1.7–63.3).

**Conclusion:**

T2HIZ is detected in more than half of refractory ZAP, and pain is more likely to remain after tSCS treatment in the T2HIZ( +) group.

## Introduction

Herpes zoster is characterized by skin rash and pain along the dermatome due to reactivation of the varicella-zoster virus [1]. The pain associated with herpes zoster is called herpes zoster-associated pain (ZAP) [2]. While most of the pain resolves spontaneously, some of the pain becomes refractory and difficult to treat [1]. ZAP can be divided into three major groups based on the duration of illness from onset, namely, as acute ZAP within the first month after onset, subacute ZAP from 1 to 3 months after onset, and chronic ZAP—also called postherpetic neuralgia (PHN)—from 3 months after onset [1, 3]. PHN is a complication of refractory neuropathic pain that occurs in 10–50% of patients with herpes zoster and significantly impacts their quality of life [1].

Treatment for herpes zoster pain depends on the time elapsed since the disease onset. For acute to subacute ZAP, nociceptive pain is the primary focus, with nonsteroidal anti-inflammatory drugs (NSAIDs) and acetaminophen administered for mild pain, and opioid analgesics for moderate to severe pain [1]. Epidural or paravertebral block with steroids may be effective as an interventional treatment when the patient is refractory to medical therapy [4–6]. In contrast, the focus in PHN is on neuropathic pain, and there are very few effective treatments. Tricyclic antidepressants, anticonvulsants, and opioids are often used as symptomatic treatments for neuropathic pain. However, these can have considerable side effects [7]. A few interventional therapies are effective in treating PHN, yet these are limited. Thus, preventing the progression of subacute ZAP to PHN remains the focus of attention [7]. Recently, among the interventional therapies, temporary spinal cord stimulation (tSCS) for subacute ZAP has been reported to be effective in preventing the transition to PHN [8–10].

At our institution, we have been administering tSCS to those patients with ZAP who were refractory to conservative treatment since 2010, with some patients responding to treatment. Most patients undergo spinal magnetic resonance imaging (MRI) before tSCS treatment at our facility to evaluate the epidural space adhesions and spinal lesions that may interfere with tSCS treatment. A previous report indicated that spinal MRI images in some patients with ZAP had T2 high-signal intensity, and more than half of them developed PHN [11]. Therefore, we hypothesized that a T2 high-signal-intensity zone (T2 HIZ) on spinal MRI would be resistant to tSCS treatment and would be a risk factor for PHN.

In this retrospective study, we selected patients who had been previously treated with tSCS at our hospital and re-examined their spinal MRIs to confirm the presence of T2 HIZ and evaluate the effect of the treatment. The purpose of this study was to examine how often a T2 HIZ is recognized in patients with subacute ZAP refractory to conservative treatment and whether it is a possible risk factor for PHN.

## Methods

This single-center, case–control study was performed at the NTT Medical Center, Tokyo, Japan. This study was approved by the ethics committee of the NTT Medical Center, Tokyo (Approval no.: 22-113). After obtaining approval from the ethics committee, the medical records of patients who underwent tSCS for ZAP between January 2010 and December 2018 were examined. This study is compliant with the STROBE guidelines for retrospective case–control studies [12].

### Patient selection

Standard indications for tSCS treatment of refractory ZAP at our institution are as follows: (i) less than 6 months after the onset of herpes zoster, (ii) insufficient analgesia with 2–3 kinds of medication, such as gabapentinoids, tricyclic antidepressants, and opioid analgesics, (iii) inadequate pain relief with 2–3 versions of nerve block therapy such as peripheral nerve blocks, epidural blocks, nerve root blocks and pulsed radiofrequency, and/or (iv) having a systemic condition that is acceptable for treatment.

For the selected patients, the exclusion criteria were: (i) patients who did not undergo spinal MRI before tSCS, (ii) patients who received tSCS at or after 91 days from the onset of herpes zoster to exclude cases that already have PHN, (iii) patients who did not have strong complaints of pain (NRS ≤ 5), and (iv) patients with an SCS treatment duration less than 7 days to filter out cases with inadequate duration of tSCS treatment.

### Magnetic resonance imaging

**Fig. 1:**
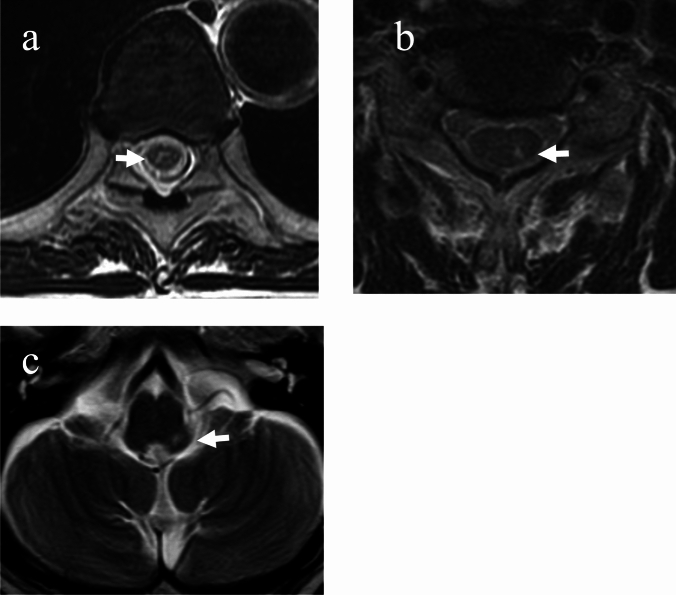
T2 HIZ of spinal cord dorsal horn in spinal MRI. Panels a, b, and c show examples of the T2 HIZ( +). White arrows indicate the lesions. **a** Thoracic spinal MRI of a patient with herpes zoster in the right T9 area. **b** Cervical spinal MRI of a patient with herpes zoster in the left C8. **c** Head MRI of a patient with herpes zoster in the left third trigeminal area. *T2 HIZ* T2 high-signal-intensity zone; *MRI* magnetic resonance imaging

Spinal MRI images were re-read by two pain clinicians and divided into two groups: T2 HIZ ( +) and T2 HIZ (−). T2 HIZ ( +) was determined when the T2 high-intensity signal area in the region of the dorsal horn of the spinal cord at the level of the responsible spinal cord was confirmed by two clinicians (Fig. 1). All MRI images were also certified by the radiologist as having no deviation in T2 HIZ ( +). Among the T2 HIZ ( +), cases in which the possibility of spinal cord signal changes caused by spinal degenerative diseases could not be ruled out were excluded from the study. T2 HIZ (−) was defined when either one of the clinicians considered that spinal MRI images showed no significant lesions in the area of the responsible spinal cord. The responsible spinal cord level was the level of the spinal cord medulla corresponding to each herpes zoster-affected spinal nerve. In the cervical nerve, the spinal nerve and spinal myelination levels are almost the same; however, the thoracic nerve has a cephalic shift of approximately 1–3 myelin segments, and the lumbar nerve has a cephalic shift of approximately 3–5 myelin segments. The two clinicians carefully verified whether the signal change at the level of the spinal cord medulla matched the spinal nerve level estimated from the skin rash.

### Patient background

The background factors related to the patient’s condition were age, sex, dermatome of the affected site, diabetes and an immunosuppressed state as background conditions, initial pain intensity measured by the NRS (numerical rating scale, 0 = no pain; 10 = the worst pain imaginable), presence of allodynia, sensation disability, and muscle weakness. Patients with no information on allodynia, sensation disability, or muscle weakness in the medical records were excluded. Progress in medical treatment was calculated based on the number of days from onset to the date of the initial visit, to spinal MRI, and the initiation of tSCS treatment. Current medications (acetaminophen, NSAIDs, tramadol, duloxetine, tricyclic antidepressants, antiepileptic drugs, and pregabalin) and interventional treatments (peripheral nerve block, nerve root block, pulsed radiofrequency, and continuous epidural block) without tSCS were investigated. Information on psychotherapy and physical therapy was not collected in the present study because ZAP is not a chronic disease and there are few cases of their provision.

### Temporary spinal cord stimulation

The standard tSCS treatment for refractory ZAP in current study is shown below. The patient was positioned supine, and the SCS lead was inserted under fluoroscopic guidance. The location of the SCS lead confirms paresthesia consistent with the affected area of the herpes zoster. Leads were anchored to the skin with thread and tape, and x-rays were used to check for misalignment of the lead position. Although it depended on the surgeon and the effect of the treatment, the stimulation period was approximately 10–20 days, and the stimulation mode was based on tonic stimulation. High-frequency or burst stimulation was used temporarily depending on device performance, changes in pain, and patient preference. As soon as the stimulation period was over, the lead was removed, and the patient is discharged. After discharge from the hospital, the patient should be visited every two weeks to several months and be treated with medications or injections, depending on the pain. The SCS stimulation period and stimulation mode were collected in the present study. Tonic stimulation is a conventional stimulation method that produces 40–60 Hz of paresthesia sensation. However, High-dose and Burst stimulations are relatively new stimulation methods characterized by paresthesia-free stimulation. High-dose stimulation comprises high-frequency stimulation at 1–10 kHz, whereas Burst stimulation comprises a series of five pulsed stimuli at 40 Hz. The stimulation mode is described as Tonic when only Tonic stimulation was used, and as Multiple stimulation when Burst or High-dose stimulation in addition to Tonic was used.

### Outcome measurement

Treatment efficacy was examined using the NRS. The NRS immediately after treatment and the final NRS were collected. The NRS immediately after treatment was the NRS at discharge after SCS extraction, and the final NRS was obtained up to 1 year later. If the outpatient visit was terminated within 1 year after treatment, the NRS at the last visit was used as the final NRS. The final NRS ≥ 3 was defined as PHN, and the rate of PHN was compared between the two groups—T2 HIZ ( +) and T2 HIZ (−). On the other hand, if the final NRS was ≤ 2, the patient was considered to have a therapeutic response to tSCS treatment.

### Statistical analysis

Statistical analyses were performed using GraphPad Prism 10 software (GraphPad Software; Inc., La Jolla, CA, USA) and EZR (Saitama Medical Center, Jichi Medical University, Saitama, Japan) which is a graphical user interface for R (The R Foundation for Statistical Computing, Vienna, Austria) [13]. Continuous variables, such as age, NRS, and number of days from onset to first visit, were tested using the Kolmogorov–Smirnov test for normality, followed by a t-test for normal distribution and the Mann–Whitney U test if not distributed normally. Sex, medication, interventional treatment, and percentage of treatment responses were determined using Fisher’s exact test. The affected dermatome was tested using the Chi-square test, followed by Bonferroni’s post-hoc test.

The effect of tSCS treatment was assessed using NRS before and after treatment and tested using one-way analysis of variance (ANOVA) followed by Bonferroni’s post-hoc test. The NRS between the T2 HIZ ( +) and T2 HIZ (−) groups were examined using two-way repeated ANOVA followed by Bonferroni’s post-hoc test. Statistical significance was set at P < 0.05.

## Results

### Patient selection

**Fig. 2:**
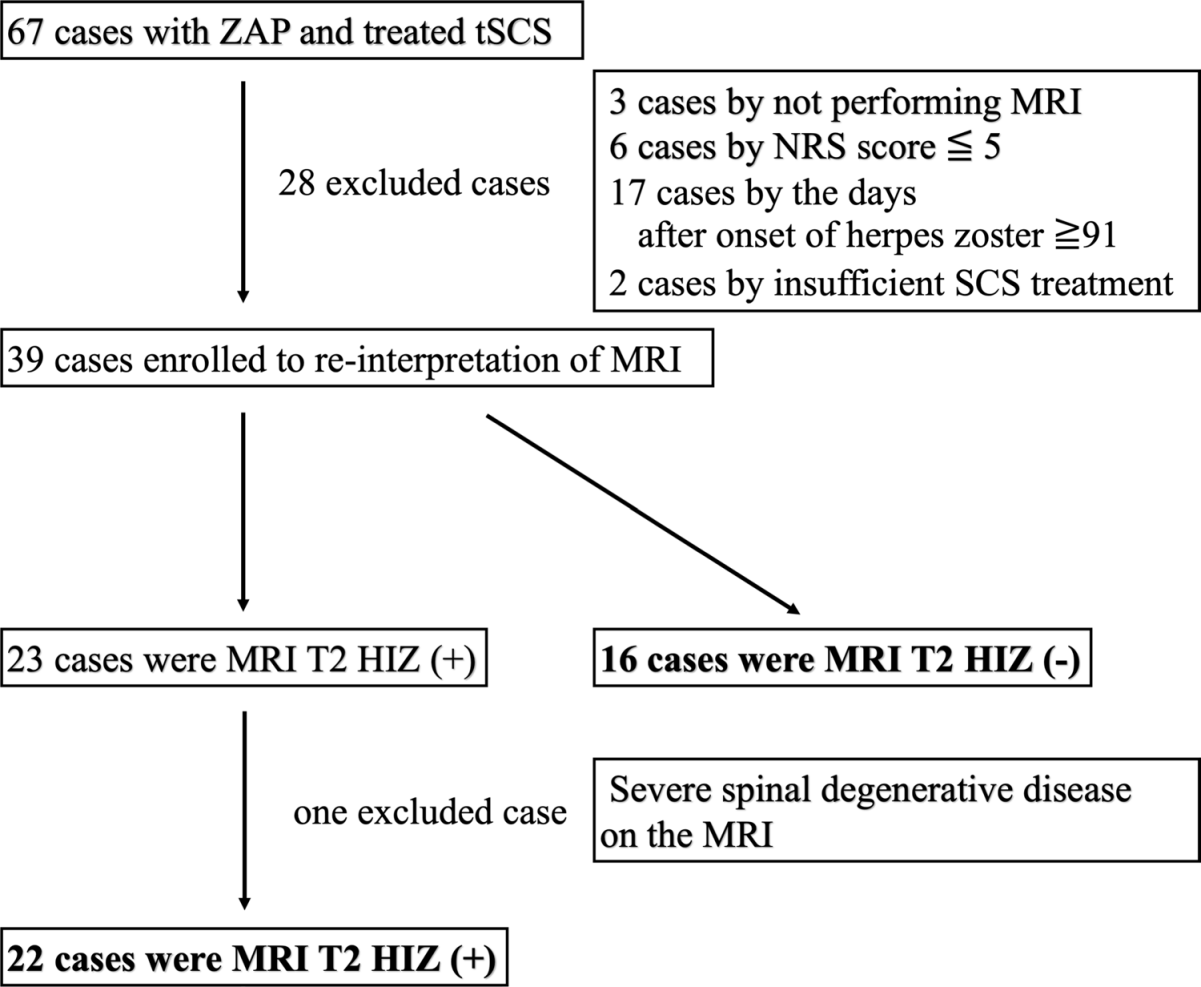
Study flowchart. *ZAP* herpes zoster associated-pain, *NRS* numerical rating scale. *T2 HIZ* T2 high-signal-intensity zone

In our database, between January 2010 and December 2018, 67 patients were identified as having undergone tSCS for ZAP. Among them, 28 patients were excluded for the following reasons: (i) spinal MRI was not performed (n = 3), (ii) more than 91 days had passed since onset (n = 17), (iii) NRS was 5 or less at baseline (n = 6), and (iv) tSCS treatment duration was less than 7 days (n = 2). Finally, 39 patients were included in this study, and their spinal MRI images were re-examined. The results of spinal MRI reinterpretation revealed that 23 patients had hyperintense lesions on T2-weighted axial MRI—T2 HIZ ( +) and 16 patients did not have the lesion—T2 HIZ (−). One of the T2HIZ ( +) cases had herpes zoster at the C4 level, and MRI scan before tSCS showed a T2 high-signal area in the dorsal horn of the spinal cord at the C3/4 level and severe spinal canal stenosis at the C4/5 level. Therefore, the possibility of signal changes associated with spinal degenerative disease could not be eliminated (Fig. 2). Hence, this case was excluded.

### Patient background

The patient backgrounds in each group are summarized in Table 1. Data of three patients with allodynia and one patient with hyposensitivity could not be found in the medical records. No significant differences were observed between T2 HIZ ( +) vs. T2 HIZ (−) groups in terms of the patients’ age, sex and NRS at the first visit. Moreover, T2 HIZ ( +) vs. T2 HIZ (−) groups were similar with regard to days from onset to first visit, MRI imaging, tSCS insertionand last visit, tSCS treatment period and mode, medications (acetaminophen, NSAIDs, tramadol and pregabalin), and interventional therapies (nerve root block, pulsed radiofrequency and continuous epidural block).

**Table 1:** Background characteristics of the two groups

	MRI T2 HIZ( +) (n = 22)	MRI T2 HIZ(−) (n = 16)	P value
Age (y)	70.2 ± 11.2	73.1 ± 9.6	0.41
Sex (Male/female)	8/14	9/7	0.32
Affected dermatome (%)			0.32
Cervical	9(41)	6 (38)	
Thoracic	12 (55)	6(38)	
Lumbar	1 (5)	3 (19)	
Sacral	0 (0)	1(6)	
Background disease			
Diabetes(%)	0/22 (0)	2/16(13)	0.16
Immunosuppression(%)	0/22 (0)	1/16 (6)	1
Initial condition			
At first visit NRS score	8.3 ± 1.3	7.8 ± 1.1	0.21
Allodynia (%)	18/22 (82)	11/13( 84)	1
Hyposensitivity (%)	18/22 (82)	11/15( 73)	0.69
Muscle weakness (%)	1/ 22(4.5)	1/16(6)	1
Days from onset			
First visit	34.5 ± 16.7	30.4 ± 17.3	0.46
MRI imaging	49.3 ± 14.3	48.4 ± 18.3	0.88
SCS administered	55.8 ± 15.9	53.4 ± 14.0	0.63
Last visit	308.4 ± 115.0	316.3 ± 139.1	0.85
tSCS treatment			
Therapy period (days)	14.0 ± 3.8	13.1 ± 3.5	0.50
Therapy mode (T/M)	15/7	12/4	0.73
Medication (%)			
Acetaminophen	10/22 (45)	5/16(31)	0.52
NSAIDs	15/22(68)	7/16 (43)	0.19
Tramadol	12/ 22 (55)	9/16(56)	1
Duloxetine	7/22 (32)	2/16(13)	0.25
Tricyclic antidepressants	15/22 (65)	9/16 (56)	0.51
Antiepileptic drugs	12/22 (55)	6/16(38)	0.34
Pregabalin	20/22 (91)	16/16(100)	0.5
Intervention (%)			
Peripheral nerve block	22/22 (100)	15/16 (94)	0.42
Nerve root block	19/22 (86)	13/16(81)	0.68
Pulsed radiofrequency	13/22 (59)	10/16(63)	1
Continuous epidural block	15/ 22 (68)	12/16(75)	0.73

*NRS* Numerical Rating Scale, *SCS* spinal cord stimulation, *T2 HIZ* T2 high-intensity zone, *MRI* magnetic resonance imaging, *NSAIDs* nonsteroidal anti-inflammatory drugs

*T* Tonic stimulation, *M* Multiple stimulation (tonic stimulation plus burst stimulation or high-dose stimulation)

### Treatment effect

**Fig. 3:**
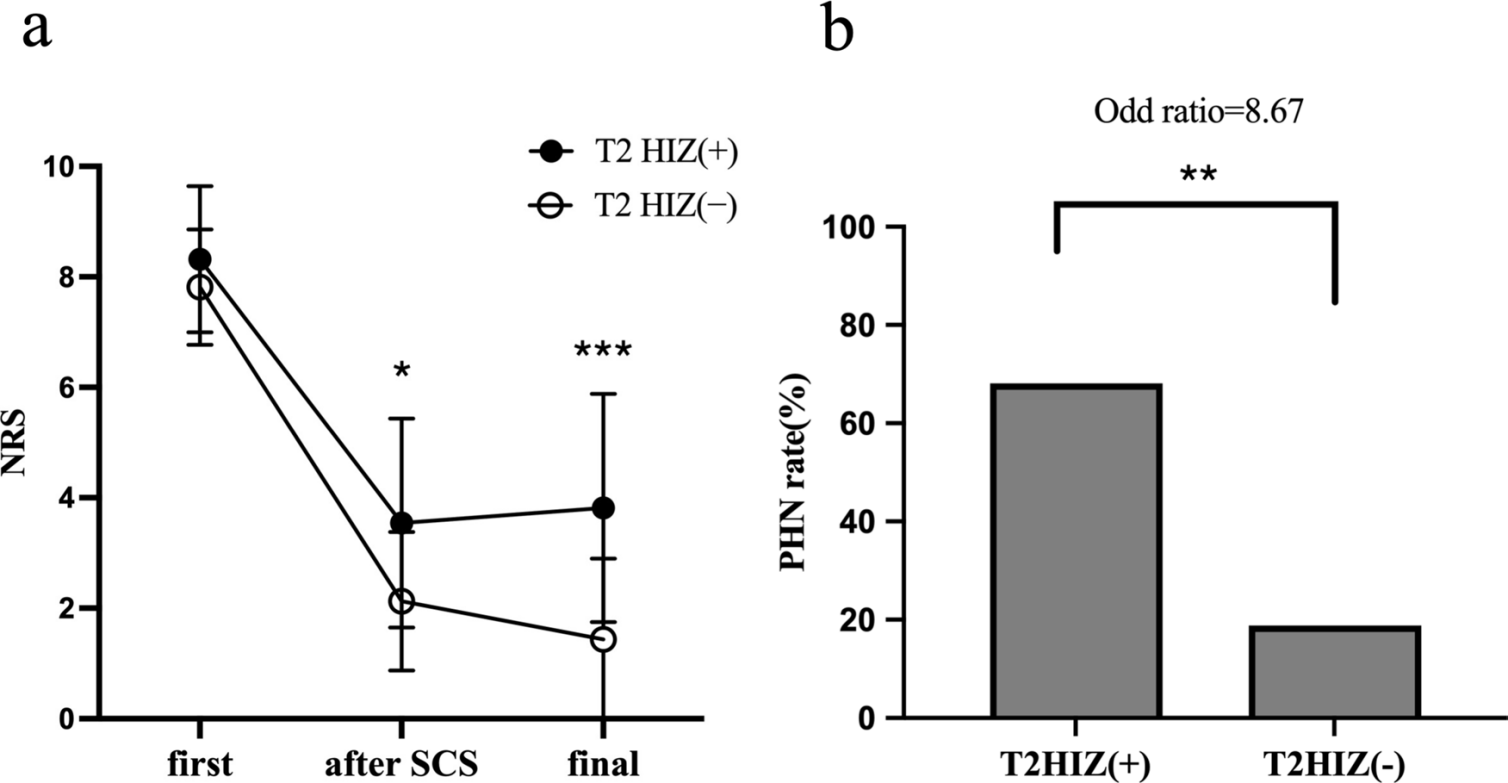
Comparison of NRS and PHN rate after tSCS between the two groups. Graph **a** shows a comparison of NRS between the T2 HIZ( +) and T2 HIZ(−) groups. The NRS at the first visit were almost similar; however, the T2 HIZ( +) group had significantly higher NRS at after SCS treatment and final visit. Graph **b** shows a comparison of PHN incidence rates after tSCS treatment between the two groups. PHN was defined as an NRS score ≥ 3 at final visit. The PHN incidence rates were 68.2% for T2 HIZ ( +) and 18.8% for T2 HIZ (−). The odds ratio of T2 HIZ ( +) for PHN was 8.67. *NRS* Numerical Rating Scale, *PHN* postherpetic neuralgia, *tSCS* temporary spinal cord stimulation, *T2 HIZ* T2 high-intensity zone, *P < 0.05, **P < 0.01, ***P < 0.001

Temporary SCS treatment significantly improved the NRS between pre- and post-treatment (pre-treatment: 8.1 ± 1.2, post-treatment: 2.9 ± 1.9; P < 0.0001), with a final treatment response rate of 20/38 (53%). Comparison of NRS during the course of treatment showed no significant difference in the pre-treatment NRS between the two groups, while the post-treatment and final NRS were significantly higher in patients from the T2HIZ ( +) group{(post-treatment:T2 HIZ ( +): 3.5 ± 1.9, T2 HIZ (−): 2.1 ± 1.2; P < 0.05), (final NRS: T2 HIZ ( +):3.8 ± 2.1, T2 HIZ (−): 1.4 ± 1.5; P < 0.001)}(Fig. 3A). Furthermore, the rate of PHN in the T2 HIZ ( +) group was significantly higher than that in the T2 HIZ (−) group (15/ 22 (68%) vs. 3/16 (19%); odds ratio = 8.67; 95% confidence interval (1.7–63.3) (Fig. 3B). The proportion of PHN corresponding to herpes zoster affected sites is shown below. The ratio of PHN in the T2 HIZ ( +) group was 6/9 (67%) in the cervical region, 8/12 (67%) in the thoracic region, and 1/1 (100%) in the lumbar region. In the T2 HIZ (−) group, the rate of PHN was 1/6 (17%) in the cervical region and 2/6 (33%) in the thoracic region.

## Discussion

In the present study, among the 38 patients with moderate-to-severe ZAP who received tSCS treatment within 90 days from onset, 22 had T2 high-intensity signal areas on spinal MRI. Temporary SCS treatment significantly improved the NRS in patients with ZAP refractory to conservative treatment. The rate of PHN after tSCS treatment was significantly higher in the T2 HIZ ( +) group. Spinal MRI T2 high-signal areas were found in more than half of patients with moderate-to-severe ZAP, and may thus be an indicator of PHN.

In the current study, 22 of 38 (58%) patients with moderate-to-severe ZAP had abnormal signals on spinal MRI. There is only one report of a T2 high-intensity region in the dorsal horn of a patient with herpes zoster. Haanpää et al. performed spinal MRI in 16 of 50 (32%) patients with ZAP, found abnormal spinal cord signals in nine patients, and reported that five of the nine (55%) patients developed PHN [11]. The higher percentage of abnormal signals on spinal MRI in this study may be attributed to the higher incidence of more severe cases than among those reported by Haanpää et al. [11]. Although this study included patients with severe herpes zoster who underwent SCS, the proportion of all patients with herpes zoster with abnormal spinal cord MRI findings is not known. The results of the current study showed that 18/38 (47%) cases remained in pain after tSCS treatment. The rate of residual pain was 15/22 (68%) in the T2 HIZ ( +) group and 3/16 (19%) in the T2HIZ (−) group. Although the frequency of PHN varies according to the patient group and the definition of PHN, it is estimated that 5.8–26% of patients with herpes zoster are affected by PHN [14–17]. The higher proportion of residual pain in the present study was attributed to a group of patients with ZAP who are refractory to other conservative treatments. Especially in the T2 HIZ ( +) group, a high proportion of patients with residual pain could be considered at risk for PHN.

Several studies have reported on the efficacy of tSCS in the treatment of subacute ZAP. Moriyama reported 14 cases of tSCS treatment for residual moderate-to-severe thoracic areas of the ZAP after continuous epidural catheterization less than 3 months after onset [9]. The VAS (range, 0–100) improved from 67.7 ± 20.2 before tSCS to 14.2 ± 14.3 after the treatment. Dong et al. described 46 cases of tSCS treatment for ZAP with an onset at < 3 months. NRS (range, 0–10) decreased from 7.28 ± 0.93 before the treatment to 3.59 ± 2.4 after treatment, and follow-up at 1 year showed improvement in the NRS value with 2 or less in 80% of cases [10]. Our results show that the overall NRS at the first visit was 8.1 ± 1.2 and improved to 2.7 ± 2.2 at the final visit. Similar to previous reports, this study demonstrated the efficacy of tSCS therapy for subacute ZAP; however, this is the first to study to demonstrate that efficacy varies based on spinal cord MRI findings.

Risk factors for PHN include older age, female sex, severe pain, severe skin rash, ocular complications in the first division of the trigeminal nerve, and an immunosuppressed state [14–17]. As this was a retrospective study, no clear information was available from medical records regarding skin rashes. In addition, the trigeminal region was not included in this study because ZAP in the affected areas was targeted for SCS treatment. However, there were no significant differences in age, sex, or pain intensity between the two groups, and there were no factors other than spinal MRI findings contributing to a difference in the response rate. Thus, abnormal spinal MRI signals may be a risk factor of PHN, as reported by Haanpää et al. [11]. If the risk factors for the development of PHN could be determined by objective evidence from MRI testing, this would be a breakthrough in the treatment of ZAP such as in the timing of tSCS introduction and the decision making process for treatment selection. Future studies should investigate whether T2 HIZ ( +) is a risk factor for PHN in all patients with herpes zoster.

Demyelination and necrosis due to severe inflammation have been reported in the dorsal horn of the spinal cord in deceased patients with severe herpes zoster [18-20]. In general, a T2 high-intensity signal on MRI is considered a finding suggestive of inflammation in the tissue. The T2 HIZ in the dorsal horn of the spinal cord observed in current study suggests that the nerve inflammation associated with herpes zoster extends into the dorsal horn of the spinal cord and could affect the severity of herpes zoster as well as risk factors for PHN.

The mechanism of temporal SCS remains unclear [21]. Conventionally, the mechanism of action of SCS is referred to as the gate-control theory; however, new stimulation methods, such as high-frequency stimulation and burst stimulation, have been added, and various theories have emerged regarding mechanism of action [22–25]. Recently, there have been reports of its effects on the dorsal horn and perineural glial cells, and methods of stimulation targeting each have been developed [26, 27]. While clinical efficacy of tSCS in ZAP has been reported, the mechanism of action remains clear. The current study’s results are similar to those of previous reports in that tSCS treatment was effective for patients with subacute ZAP. However, patients in the T2 HIZ ( +) group tended to have slightly more residual pain with mainly tonic stimulation being used in the present study. In the future, SCS treatment may have potential to be a more effective treatment for ZAP with T2 HIZ ( +) using a new stimulation method or early therapeutic intervention.

There are several limitations in this study. First, this was a single-center, retrospective study with a few cases. The small number of cases made it difficult to perform multivariate analysis considering risk factors. Second, long-term follow-up was not available, and some patient information was lacking owing to the retrospective nature of the study. In particular, the absence of NRS assessment prior to tSCS treatment may affect the evaluation of tSCS treatment efficacy, and psychological evaluations such as the pain catastrophizing scale (PCS) or the Hospital Anxiety and Depression scale (HADS) may provide inadequate evaluation of chronic pain. Therefore, prospective studies are warranted. Third, the diagnosis for T2 HIZ based on MRI has not been established. In the present study, the radiologist also confirmed the results, and there is confidence regarding T2 HIZ ( +), whereas there were some cases among T2 HIZ (−) for which opinions were divided. The MRI in current study was not performed for the purpose of evaluating T2HIZ, so the conditions were not sufficient for T2 HIZ diagnosis. In the future, it will be necessary to establish optimal MRI conditions and accumulate knowledge about MRI T2 high-signal-intensity lesions.

In conclusion, T2 HIZ was detected in more than half of cases with refractory ZAP, and pain was more likely to remain after tSCS treatment in the T2 HIZ ( +) group. Further studies are needed to determine whether T2HIZ is a risk factor for PHN.

## Data Availability

The data supporting this study’s findings are available from the corresponding author upon reasonable request.

## References

[1] Cohen JI. Clinical practice: herpes zoster. N Engl J Med. 2013;369:255–63. 23863052 10.1056/NEJMcp1302674 PMC4789101

[2] Dworkin RH, Gnann JW Jr, Oaklander AL, Raja SN, Schmader KE, Whitley RJ. Diagnosis and assessment of pain associated with herpes zoster and postherpetic neuralgia. J Pain. 2008;9:S37–44. 18166464 10.1016/j.jpain.2007.10.008

[3] Dworkin RH, Portenoy RK. Proposed classification of herpes zoster pain. Lancet. 1994;343:1648. 7911959 10.1016/s0140-6736(94)93106-2

[4] van Wijck AJ, Opstelten W, Woons KG, van Essen GA, Stolker RJ, Kalkman CJ. The PINE study of epidural steroids and local anaesthetics to prevent postherpetic neuralgia: a randomised controlled trial. Lancet. 2006;367:219–24. 16427490 10.1016/S0140-6736(06)68032-X

[5] Kumar V, Krone K, Mathieu A. Neuraxial and sympathetic blocks in herpes zoster and postherpetic neuralgia: an appraisal of current evidence. Reg Anesth Pain Med. 2004;29:454–61. 15372391 10.1016/j.rapm.2004.04.010

[6] Ji G, Niu J, Shi Y, Hou L, Lu Y, Xiong L. The effectiveness of repetitive paravertebral injections with local anesthesics and steroids for the prevention of postherpetic neuralgia in patients with acute herpes zoster. Anesth Analg. 2009;109:1651–5. 19713253 10.1213/ANE.0b013e3181b79075

[7] Jeon YH. Herpes zoster and postherpetic neuralgia: practical consideration for prevention and treatment. Korean J Pain. 2015;28:177–84. 26175877 10.3344/kjp.2015.28.3.177 PMC4500781

[8] Iseki M, Morita Y, Nakamura Y, Ifuku M, Komatsu S. Efficacy of limited-duration spinal cord stimulation for subacute postherpetic neuralgia. Ann Acad Med Singap. 2009;38:1004–6. 19956824

[9] Moriyama K. Effect of temporary spinal cord stimulation on postherpetic neuralgia in the thoracic nerve area. Neuromodulation. 2009;12:39–43. 22151221 10.1111/j.1525-1403.2009.00186.x

[10] Dong DS, Yu X, Wan CF, Liu Y, Zhao L, Xi Q, Cui WY, Wang QS, Song T. Efficacy of short-term spinal cord stimulation in acute/subacute zoster-related pain: a retrospective study. Pain Physician. 2017;20:E633–45. 28727708

[11] Haanpää M, Dastidar P, Weinberg A, Levin M, Miettinen A, Lapinlampi A, Laippala P, Nurmikko T. CSF and MRI findings in patients with acute herpes zoster. Neurology. 1998;51:1405–11. 9818869 10.1212/wnl.51.5.1405

[12] von Elm E, Altman DG, Egger M, Pocock SJ, Gøtzsche PC, Vandenbroucke JP, STROBE Initiative. The Strengthening the Reporting of Observational Studies in Epidemiology (STROBE) statement: guidelines for reporting observational studies. Lancet. 2007;370:1453–7. 18064739 10.1016/S0140-6736(07)61602-X

[13] Kanda Y. Investigation of the freely available easy-to-use software ‘EZR’ for medical statistics. Bone Marrow Transplant. 2013;48:452–8. 23208313 10.1038/bmt.2012.244 PMC3590441

[14] Forbes HJ, Bhaskaran K, Thomas SL, Smeeth L, Clayton T, Mansfield K, Minassian C, Langan SM. Quantification of risk factors for postherpetic neuralgia in herpes zoster patients: a cohort study. Neurology. 2016;87:94–102. 27287218 10.1212/WNL.0000000000002808 PMC4932239

[15] Forbes HJ, Thomas SL, Smeeth L, Clayton T, Farmer R, Bhaskaran K, Langan SM. A systematic review and meta-analysis of risk factors for postherpetic neuralgia. Pain. 2016;157:30–54. 26218719 10.1097/j.pain.0000000000000307 PMC4685754

[16] Wei S, Li X, Wang H, Liu Q, Shao L. Analysis of the risk factors for postherpetic neuralgia. Dermatology. 2019;235:426–33. 31256167 10.1159/000500482

[17] Zhou H, Wang Z, Jin H, Chen X, Lei L. A systematic review and meta-analysis of independent risk factors for postherpetic neuralgia. Ann Palliat Med. 2021;10:12181–9. 35016460 10.21037/apm-21-3028

[18] Hogan E, Krigman M. Herpes zoster myelitis. Evidence for viral invasion of spinal cord. Arch Neurol. 1973;29:309–13. 4355263 10.1001/archneur.1973.00490290049004

[19] Devinsky O, Cho E, Petito C. Herpes zoster myelitis. Brain. 1991;114:1181–96. 1648419 10.1093/brain/114.3.1181

[20] Moshayedi P, Thomas D, Rinaldo C. Subacute histopathological features in a case of varicella zoster virus myelitis and post-herpetic neuralgia. Spinal Cord Ser Cases. 2018;4:4–7. 10.1038/s41394-018-0068-5 PMC5884843 29707236

[21] Jensen MP, Brownstone RM. Mechanisms of spinal cord stimulation for the treatment of pain: still in the dark after 50 years. Eur J Pain. 2019;23:652–9. 30407696 10.1002/ejp.1336 PMC6491991

[22] Chakravarthy K, Fishman MA, Zuidema X, Hunter CW, Levy R. Mechanism of action in burst spinal cord stimulation: review and recent advances. Pain Med. 2019;20:S13-22. 31152180 10.1093/pm/pnz073 PMC6544550

[23] Linderoth B, Foreman RD. Conventional and novel spinal stimulation algorithms: hypothetical mechanisms of action and comments on outcomes. Neuromodulation. 2017;20:525–33. 28568898 10.1111/ner.12624

[24] Rock AK, Truong H, Park YL, Pilitsis JG. Spinal cord stimulation. Neurosurg Clin N Am. 2019;30:169–94. 30898269 10.1016/j.nec.2018.12.003

[25] Sdrulla AD, Guan Y, Raja SN. Spinal cord stimulation: clinical efficacy and potential mechanisms. Pain Pract. 2018;18:1048–67. 29526043 10.1111/papr.12692 PMC6391880

[26] Morales A, Yong RJ, Kaye AD, Urman RD. Spinal cord stimulation: comparing traditional low-frequency tonic waveforms to novel high frequency and burst stimulation for the treatment of chronic low back pain. Curr Pain Headache Rep. 2019;23:25. 30868285 10.1007/s11916-019-0763-3

[27] Wang ZB, Liu YD, Wang S, Zhao P. High-frequency spinal cord stimulation produces long-lasting analgesic effects by restoring lysosomal function and autophagic flux in the spinal dorsal horn. Neural Regen Res. 2022;17:370–7. 34269212 10.4103/1673-5374.317989 PMC8463971

